# Association of dietary intake and cervical cancer: a prevention strategy

**DOI:** 10.1186/s13027-023-00517-8

**Published:** 2023-07-06

**Authors:** Elham Nazari, Malihe Hasanzadeh, Reza Rezvani, Marzieh Rejali, Mohaddeseh Badpeyma, Zeinab Delaram, Leila Mousavi-Seresht, Mahdieh Akbari, Majid Khazaei, Gordon A. Ferns, Amir Avan

**Affiliations:** 1 Metabolic Syndrome Research Center, Mashhad University of Medical Sciences, Mashhad, Iran; 2 College of Medicine, University of Warith Al-Anbiyaa, Karbala, Iraq; 3 Department of Gynecology Oncology, Woman Health Research Center, Faculty of Medicine, Mashhad University of Medical Sciences, Mashhad, Iran; 4 Department of Medical Nutrition, Faculty of Medicine, Mashhad University of Medical Sciences, Mashhad, Iran; 5 Student Research Committee, Department of Clinical Nutrition, Nutrition Research Center, School of Nutrition and Food Sciences, Tabriz University of Medical Sciences, Tabriz, Iran; 6 Department of Computer Science, The University of Texas at Dallas, Richardson, USA; 7 Division of Medical Education, Brighton and Sussex Medical School, Falmer, Brighton, Sussex UK; 8 School of Biomedical Sciences, Faculty of Health, Queensland University of Technology, Brisbane, Australia

**Keywords:** Cervix cancer, HPV, Nutrition, Dietary, Vitamin, Factor affecting, Machine learning, Correlation, Prevention

## Abstract

**Introduction:**

Cervical cancer is one of lethal cancers in women. As a global concern, identifying important factors of cancer is a useful strategy for prevention. Due to the role of diet/nutrition factors for cancer, the purpose of our study was to determine the impact of 150 nutrition/vitamin factors and 50 non-nutritional factor in cervical cancer and phase.

**Methods:**

Population samples of 2088 healthy subjects and patients with cervical cancer were investigated. 200 factors such as vitamin E, B1, B6, fruits, HPV, and age were gathered. Deep learning, Decision tree, and correlation matrix were used for modeling and identifying important factors. SPSS 26, R4.0.3, and Rapid miner were utilized for implementation.

**Results:**

Our findings indicated that zinc, Iron, Niacin, Potassium, Phosphorous, and Cooper have a beneficial impact in reducing the risk of cervical cancer and progression of phase in Iranian women, as well as Salt, snacks and milk Were identified as high-risk food factors (*P* value < 0.05 and coefficient correlation > 0.6). Also, alcohol, and sex patient with two groups, HPV positive have an impact on cervical cancer incidence. Phosphorus and selenium in the Micronutrients category (R^2^ = 0.85, AUC = 0.993) and polyunsaturated fatty acid and salt in the Macronutrients category and other categories of nutrients were identified as the most effective factors in cervical cancer using deep learning (R^2^ = 0.93, AUC = 0.999).

**Conclusions:**

A diet and rich nutrition can be helpful for the prevention of cervix cancer and may reduce the risk of disease. Additional research is necessary for different countries.

## Introduction

Cervical cancer is the third most common gynecological cancer in young women and the leading cause of mortality [1–3] every 2 min, one woman dies from cervix cancer [4]. 85% of new cases are in developing countries [5]. Cervical cancer is one of the disastrous menaces to women’s lives. Most of them have not been diagnosed [6]. In recent years cervical cancer has been a concern to primary physicians [7]. This is cancer, a largely preventable disease [8]. Therefore, the Development of national control and prevention program for cervical cancer should be considered to decrease cervical cancer incidence, morbidity and mortality, improve the quality of care and cost reduction [9]. Identifying the factors that affect cancer is an important prevention strategy. Many studies have pointed out the role of human papillomavirus (HPV) as a necessary cause of cervical cancer [10, 11]. Marriage age, marital status, age of first pregnancy, smoking, family history, multiparty, Education level and Economical status, also are mentioned in other studies [12]. In recent Research, dietary antioxidants, such as vitamins A, C, D, E and nutrition hold a rather great share in cervical cancer prevention [13–16]. There is growing evidence related to the effect of nutrients on cancer prevention. On the other hand, because there are geographical differences in the incidence, risk factors and mortality of cervical cancer, studies in different countries are necessary [12, 16]. Considering the lack of a comprehensive study related to many aspects of dietary/nutritional factors on Cervical cancer, the current study is mainly aimed to investigate the importance of 91 nutritious and vitamin factors also 31 demographic, sexual factor and medical examination factors on the development of cervical cancer and then formulate a strategy for prevention.

We considered three objectives for meeting the main purpose of the study:

*Aim1* Regarding to the importance of diet/nutrition in cervical cancer prevention, the correlation between all dietary/nutritional variables on cervical cancer and phases were studied and the preventive and reductive effects of the nutritional intake on cervical cancer, HPV and phases were reported.

*Aim2* Due to the importance of HPV and sexually related factors on cervical cancer incidence, the binary correlation of these variables on cervical cancer and phases were examined using a correlation matrix.

*Aim3* The effect coefficient of each category of variables such as macronutrients, micronutrients and junk foods on cervical cancer were calculated using deep learning and decision tree models.

## Material and methods

### Participants and data source

This was a population-based study involving 2088 Iranian cases (Mashhad). All participants gave informed, written consent to contribute to the survey, reviewed and approved by the ethics committee of Mashhad University of Medical Sciences (MUMS). A semi-quantitative Food Frequency Questionnaire (FFQ) was used in the clinic to assess dietary habits. The FFQ took roughly 40 min to complete and collected data on 65 different food products. To reduce estimating errors in portion and consumption frequency, the FFQ was administered by specialized nutritionists via face-to-face interview. For the previous year, the frequency of consumption of different food products was recorded on a daily, weekly, monthly, rarely, and never basis. The reference serving size was applied to determine portion sizes. Food items were categorized into 17 food groups, including fast food, fruits, and vegetables. The total energy intake was calculated by adding all of the food energy intakes together. 200 factors were collected from samples. 91 nutritional factors and 31 non-nutritional factors, were identified as suitable for modeling based on expert opinion in two rounds of the Delphi method, the descriptions of variables can be seen in Table 1.

**Table 1:** Variables used in the study

Category	Subcategory	Category	Subcategory
*Nutritional variables*			
Micronutrient	Sodium (mg)	Macronutrient	Carbohydrate (g)
	Potassium (mg)		Protein (g)
	Calcium (mg)		Fat (g)
	Magnesium (mg)		Fiber (g)
	Phosphorus (mg)		Carbohydrate (g)
	Iron (mg)		
	Copper (mg)	Carbohydrates	Glucose (g)
	Zinc (mg)		Fructose (g)
	Chloride (mg)		Sucrose (g)
	Manganese (mg)		Maltose (g)
	Selenium (mg)		Lactose (g)
	Lodine (mg)		
	Retinol (mg)	Fat	Saturated fat (g)
	Carotene (mg)		Monounsaturated fatty acid (MUFA)(g)
	Vitamin D(mg)		Polyunsaturated fatty acid(PUFA)(g)
	Vitamin E (mg)		Trans fatty acids (g)
	Thiamin (mg)		Cholesterol (g)
	Riboflavin (mg)		
	Niacin (mg)		
	Vitamin B6 (mg)	Meat	Egg
	Vitamin B12 (mg)		Fish
	Folate (mg)		Tuna
	Panthotene acid(mg)		Shrimp
	Biotine(mg)		Lamb meat
	Vitamin C (mg)		Veal meat
			Feral meat
Cereals	White bread		Poultry
	Spaghetti		Viscera Meat
	Rice		Nuts
			Legumes
Junk food	Snacks		
	Oil frying food		
	Biscuit	Vegetables	Potato
	Cake		Spinach
	Pizza		Vegetable
	Sauce		Tomato
	Chips		Cucumber
	Chocolate		lettuce
	Carbonated drinks		Garlic and onion
	Delster(non-alcoholic Beer)		
Beverage	Tea		
	Coffee	Dairy	Milk
	CoffeeMilk		Yogurt
	Water		Cheese
			Cream
Fruits	Trees fruit		IceCream
	Seasonal fruit		Doogh(Iranian soft drink)
	Fruit juice		
	Dried fruit (Nuts)	High calorie	Honey
			Sugar
Tot.N2.g	Tot.sug.mg	Starch	Salt
*Non-nutritional variables*			
Demographic	Age	Medical examination factor	Exocervix
	Marriage		Wart
	Smoking		Smear
	Alcohol		Hpv positive
	Residient_place		HPV_sign _cat
	Weight		
	Education_status		
	Financial_status		
	Residient_place		
Sexual related factors	Sexmate_patient_2group	Treatment	Doing vaccination
	Age_first_pregnancy		Treatment_method
	Number of sex in a month		
	Natural_delivery vs Cesarean	Target variables	Case_control(Cancer/healthy patient)
	Menstrual disorder		Phase
	Age_firstsex		
	Number_Sexmate_for_spouse		
	Delivery		
	Successful_pregnancy		
	Number_Cesarean_delivery		
	Contraception_method		
	Age_first_pregnancy		
	Abortion		

### Statistical analysis

Descriptive analysis, normality test (Kolmogorov–Smirnov tests) and Spearman correlation were performed by SPSS 26. A significance level of 0.05 was considered for analysis.

### Machine learning methods

Deep learning and decision tree models were used to identify the effective factors in a category of variables including macronutrients and micronutrients. The significant variables gained from the feature selection method (Weight by Correlation) were the final parameters in creating the model. The two machine learning techniques used in the study, the decision tree and deep learning, are described following. Also the present study used the correlation matrix to investigate the dependence between variables. A correlation matrix depicts the coefficient of correlation between variables. The correlation coefficient is measured from − 1 to 1. A positive correlation points that the variables are in the same direction, while a negative correlation shows the variables in opposite directions. The lack of correlation is displayed by 0.

#### Decision tree

The Decision tree is a very popular class of predictive models due to their interpretability and best performance special on categorical data. It is a tree-based technique in which a data separating sequence characterizes any path from the root node to the leaf until a Boolean outcome is obtained. Decision trees are an effective tool that may be utilized in various domains, including machine learning, image processing, and pattern recognition [17].

#### Deep learning

Deep learning is part of machine learning methods based on artificial neural networks. Deep learning allows computational models with several layers to learn multiple degrees of abstraction for data representations and can automatically learn feature selection from many varying data, Deep learning uses the backpropagation algorithm to show how a machine should adjust its internal parameters that are used to compute the representation in each layer from the representation in the previous layer, revealing intricate structure in massive data sets [18].

#### Computational workflow

R 4.0.3 and Rapid miner version 9.10 were utilized for modeling. For Decision tree modeling, the max depth = 10, minimal gain = 0.01, minimal leaf size = 2, minimal size for split = 4, number of pre-pruning alternatives = 3, confidence = 0.1 and were some of the tuning hyper parameters which were considered. In deep learning, parameters of epochs = 20, activation function = Rectifier and learning rate = 0.01 were set.

The standard workflow was utilized to create, evaluate, and optimize methods explained as follows.

##### Splitting data into training and test sets

To provide some independent evaluation levels, it is common practice to split the source data set into two parts: training and test data. The model is then optimized using the training data and independently evaluated using the test data.

##### Performance measures optimization and generalized predictive ability

In the current study, 70/30 train/test ratios were determined for machine learning models. For each workflow, a model with the fixed optimal hyper parameter values is retrained on data and randomly sampled from the complete data set, and then evaluated on the unused data.

##### Model evaluation using a test set

Machine-learning methods assessment was performed by 5 indicators, including the accuracy, R^2^, MSE, and AUC.

$${\text{Accuracy}} = \left( {{\text{TP}} + {\text{TN}}} \right)/\left( {{\text{TP}} + {\text{TN}} + {\text{FP}} + {\text{FN}}} \right)$$

where TP—true positive; FP—false positive; TN—true negative; FN—false negative.

$${\text{MSE}}\;({\text{Mean Squared Error}}) = \left( {1/{\text{n}}} \right)*\Sigma \left( {{\text{actual}} - {\text{forecast}}} \right)2$$

where Σ—a symbol that means “sum”; n—sample size; actual—the actual data value; forecast—the predicted data value.

R^2^ (R-Squared) = 1 − unexplained variation/total variation. It is the coefficient of determination and tells you the percentage variation in y explained by x-variables.

AUC (area under the curve)*:* It represents the degree of separability. It illustrates how much the capability of the model in distinguishing between classes.

## Results

### Data description

Table 2 shows the mean and standard deviation of the quantitative variables. The Frequency and percentage of cancer patients and healthy people participating in the study are also mentioned.

**Table 2:** Characteristic of population

Attributes	Cancer patients	Healthy people
	SD ± Mean	SD ± Mean
Age	34.82 ± 9.81	44.26 ± 5.65
weight	61.12 ± 20.95	70.19 ± 12.77
Wart_history	4.18 ± 18.24	
Age_first sex	19.38 ± 6.38	
Number_sex_in_month	5.38 ± 4.74	
Number_sexmate _for_spouse	1.58 ± 1.41	
Age_first_pregnancy	26.56 ± 11.50	
Number_delivery _abortion	0.86 ± 1.53	
Natural_delivery	1.06 ± 1.50	
Number_cesarean_delivery	0.4 ± 0.73	
	**Frequency (%)**	
Phase		
Phase1	564 (27)	999 (47.8)
Phase2	525 (25.2)	
Marriage		
Single	46 (2.2)	
Married	943 (45.2)	
Divorced	84 (4)	
Widow	11 (0.5)	
Missing	1004 (48.1)	
Education_status		
Illiterate	32 (1.5)	
Less than diploma	504 (24.2)	
More than diploma	548 (26.2)	
Missing	1004 (48.1)	
Smoking		
Not smoking	1501 (71.8)	
Ex-smoking	119 (5.7)	
Currently	310 (14.9)	
Passive smoking	158 (7.6)	
HPV_positive		
Negative	64 (3.1)	1560 (74.7)
HPV+	464 (22.2)	
HPV_sign_cat		
All LR	118 (5.7)	1546 (74)
HR + LR	121 (5.8)	
All + HR	255 (12.2)	
Negative	48 (2.3)	
Exocervix		
CIN1	475 (22.7)	1454 (69.6)
CIN2	72 (3.4)	
CIN3	49 (2.5)	
Cancer	38 (1.8)	
Wart		
No wart	453 (21.7)	1591 (76.2)
Genital wart	44 (2.1)	
Alcohol		
At all	919 (44)	
Quit (in the past)	71 (3.4)	
Currently	78 (3.7)	
Missing	1020 (48.9)	
Menstrul_disorder		
Yes	346 (16.6)	
No	663 (31.8)	
Missing	1079 (51.6)	
Doing vaccination		
Yes	248 (11.9)	
No	758 (36.3)	
Missing	1082 (51.8)	
Residient_place		
City	1020 (48.9)	
Rural	67 (3.2)	
Missing	1001 (47.9)	
Financial_status		
Very poor	9 (0.4)	
Poor	300 (14.4)	
Moderate	706 (33.8)	
Rich	68 (3.3)	
Missing	1005 (48.1)	
	1089 (0.52%)	1000(0.48%)

### Data analytics

The results of data analysis can be seen in the following text:

#### Response to the aim1: the correlation between the dietary/nutritional intake and the risk of cervical cancers and phase progression

Tables 3 and 4 show the correlation between dietary/nutritional intake and the risk of cervical cancers and phase progression.

**Table 3:** The preventive and reductive effects of the dietary/nutritional intake on cervix cancer

Variable1	Variable2		Correlation coefficients	Type of correlation
Cervix Cancer	Micronutrients	Vitamin E	− 0.730	Preventive and reductive diets/nutrients (strong)
		Zinc	− 0.678	Preventive and reductive diets/nutrients (strong)
		Iron	− 0.671	Preventive and reductive diets/nutrients (strong)
		Phosphorus	− 0.652	Preventive and reductive diets/nutrients (strong)
		Vitamin B3(Niacin (mg))	− 0.648	Preventive and reductive diets/nutrients (strong)
		Vitamin B6	− 0.602	Preventive and reductive diets/nutrients (strong)
		Cooper	− 602	Preventive and reductive diets/nutrients (strong)
		Potassium	− 0.574	Preventive and reductive diets/nutrients (moderate)
		Thiamin	− 0.558	Preventive and reductive diets/nutrients (moderate)
		Folate	− 0.526	Preventive and reductive diets/nutrients (moderate)
		Calcium	− 0.623	Preventive and reductive diets/nutrients (strong)
	Macronutrients	Protein	− 0.637	Preventive and reductive diets/nutrients (strong)
		Dietary Fiber	− 0.568	Preventive and reductive diets/nutrients (strong)
	Fruits	Tree fruit	− 0.745	Preventive and reductive diets/nutrients (strong)
		Seasonal fruit	− 0.740	Preventive and reductive diets/nutrients (strong)
	Dairy	Yogurt	0.778	High-risk diets/nutrients (strong)
		Milk	0.775	High-risk diets/nutrients (strong)
	Junk food	Snack	0.663	High-risk diets/nutrients (strong)
	Fat	Polyunsaturated fatty acid	− 0.723	Preventive and reductive diets/nutrients (strong)
		Tot.N2.g	− 0.642	Preventive and reductive diets/nutrients (strong)
		Salt	0.784	High-risk diets/nutrients (strong)
		Starch	− 0.569	Preventive and reductive diets/nutrients (moderate)

**Table 4:** The preventive and reductive effects of the dietary/nutritional intake on phase progression

Variable1		Variable2	Correlation coefficients	Type of correlation
Phase	Micronutrients	Manganese	− 0.80	Preventive and reductive diets/nutrients (strong)
		Magnesium	− 0.743	Preventive and reductive diets/nutrients (strong)
		Vitamin B3	− 0.744	Preventive and reductive diets/nutrients (strong)
		Cooper	− 0.731	Preventive and reductive diets/nutrients (strong)
		Zinc	− 0.731	Preventive and reductive diets/nutrients (strong)
		Folate	− 0.729	Preventive and reductive diets/nutrients (strong)
		Potassium	− 0.725	Preventive and reductive diets/nutrients (strong)
		Phosphorus	− 0.713	Preventive and reductive diets/nutrients (strong)
		Vitamin B1	− 0.695	Preventive and reductive diets/nutrients (strong)
		Biotin	− 0.689	Preventive and reductive diets/nutrients (strong)
		Iron	− 0.678	Preventive and reductive diets/nutrients (strong)
		Selenium	− 0.673	Preventive and reductive diets/nutrients (strong)
	Macronutrients	Protein	− 0.8	Preventive and reductive diets/nutrients (strong)
		Dietary Fiber	− 0.798	Preventive and reductive diets/nutrients (strong)
	Vegetables	Onion and garlic	− 0.737	Preventive and reductive diets/nutrients (strong)
		Tomato	− 0.754	Preventive and reductive diets/nutrients (strong)
		Cucumber	− 0.749	Preventive and reductive diets/nutrients (strong)
		Salad	− 0.744	Preventive and reductive diets/nutrients (strong)
		Vegetable	− 0.733	Preventive and reductive diets/nutrients (strong)
	Fruits	Tree fruit	− 0.755	Preventive and reductive diets/nutrients (strong)
		Seasonal fruit	− 0.736	Preventive and reductive diets/nutrients (strong)
	High calorie	Honey	− 0.607	Preventive and reductive diets/nutrients (strong)
	Dairy	Cheese	− 0.735	Preventive and reductive diets/nutrients (strong)
	Meat	Legume	− 0.754	Preventive and reductive diets/nutrients (strong)
		Mutton	− 0.729	Preventive and reductive diets/nutrients (strong)
		Poultry	− 0.715	Preventive and reductive diets/nutrients (strong)
		Veal meat	− 0.556	Preventive and reductive diets/nutrients (moderate)
	Cereal	Rice	− 0.788	Preventive and reductive diets/nutrients (strong)
	Fat	Polyunsaturated fatty acid	− 0.798	Preventive and reductive diets/nutrients (strong)
	Beverage	Tea	− 0.745	Preventive and reductive diets/nutrients (strong)
	Other nutrient factors	Tot.N2.g	− 0.80	Preventive and reductive diets/nutrients (strong)
		Starch	− 0.798	Preventive and reductive diets/nutrients (strong)
		Tot.sug	− 0.798	Preventive and reductive diets/nutrients (strong)

Note that the correlation coefficient less than 0.3 is considered weak, the coefficient between 0.3 and 0.6 is moderate and the coefficient greater than 0.6 is considered strong. Significant values with medium and high coefficients were listed in Table3. Positive numbers indicate high-risk diets/nutrients and negative numbers indicate Preventive and Reductive diets/nutrients effect.

**Fig. 1:**
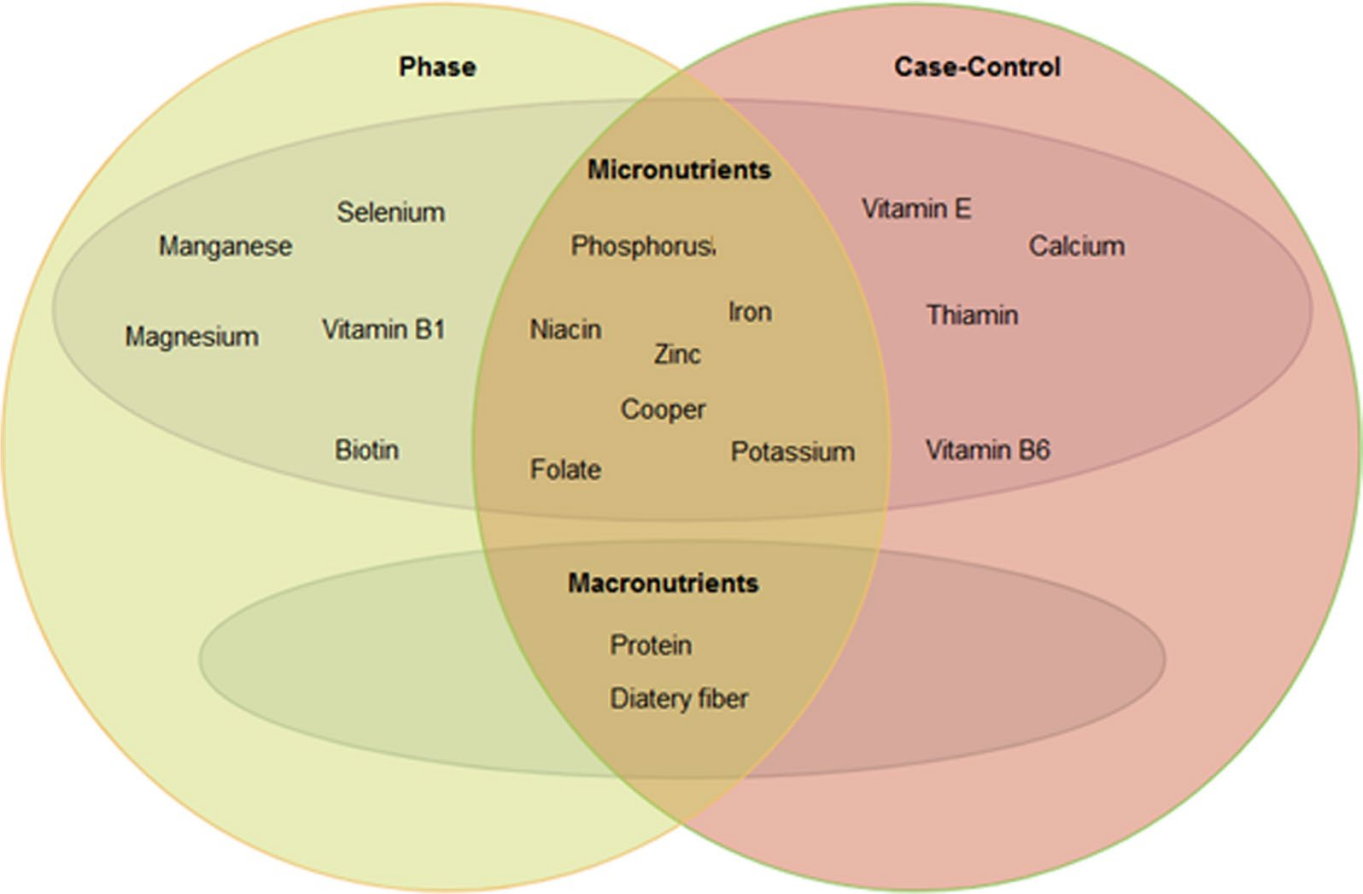
Important macronutrients and micronutrients affecting cervix cancer and phase

The findings of Tables 3 and 4 revealed that zinc, iron, niacin, potassium, phosphorous, cooper and folate have an impact in reducing the risk of cervical cancer and progression of phase (see Fig. 1), as well as salt, snack and milk were identified as high risk factors. Dietary fiber, starch and Tot.N2.g also has a beneficial impact on cervical cancer and phase. In Fig. 1 is displayed important macronutrients and micronutrients affecting on cervical cancer and its phases. Seasonal and tree fruits also have a good effect on cancer and phase, Meat and vegetables as well have a reducing effect on phase progression.

#### Response to aim2: correlation of HPV and sexually related factors with cervix cancer and phases

**Fig. 2:**
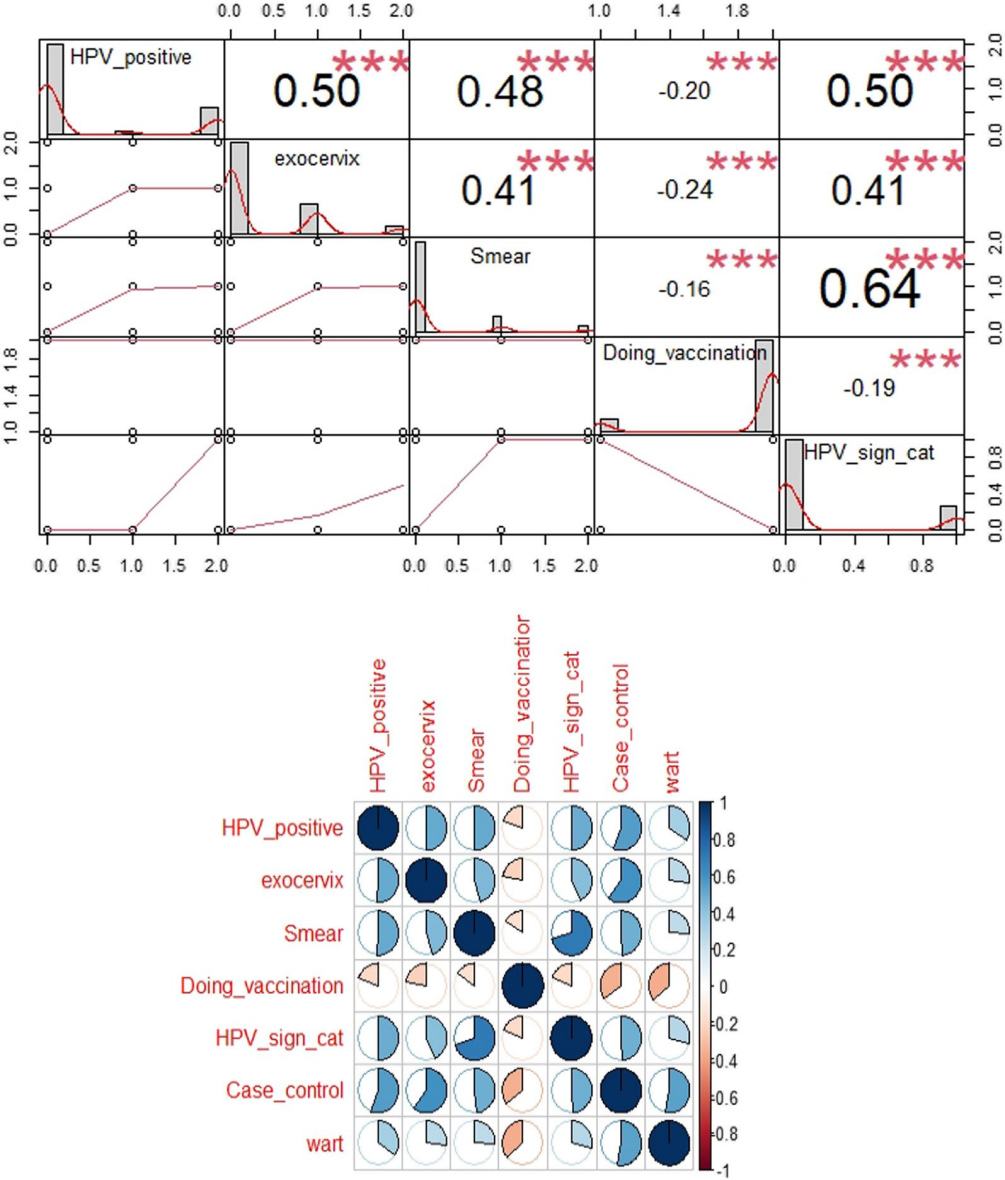
The correlation between crucial medical examination and cervical cancer

**Fig. 3:**
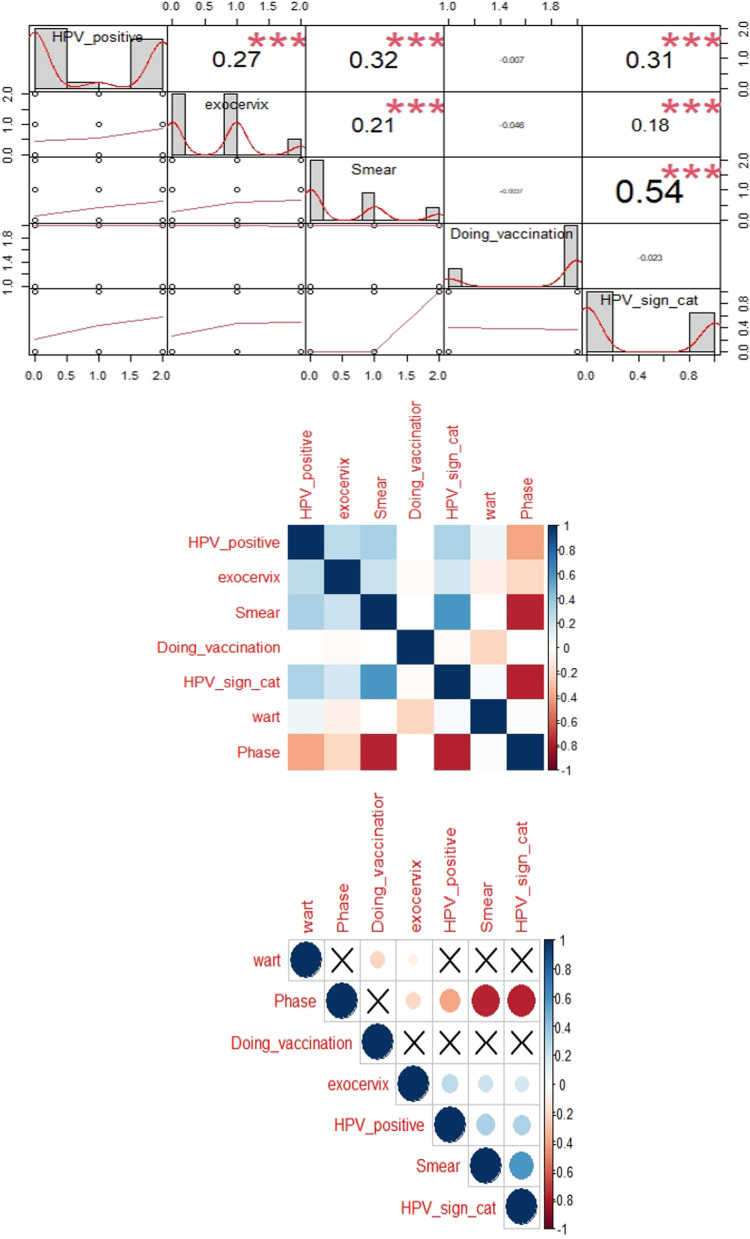
The correlation between phase and crucial medical examination

Using the correlation matrix, we examined the dual correlation between cervical cancer/phase with crucial medical examination variables. The results are shown in Figs. 2 and 3.

##### The correlation between crucial medical examination and cervical cancer

Figure 2 showed that Data is not normally distributed and a high coefficient was detected between cervix and exocervix, hpv_positive, smear, wart and hpv_sign_cat in positive direction.

##### The correlation between crucial medical examination and phase

Figure 3 showed that Data is not normally distributed and the high coefficient was detected between phase and HPV_sign_cat and smear in negative direct.

Correlation between sexual factors and demographic on cervical cancer can be seen following (Tables 5 and 6).

**Table 5:** Correlation between sexually related variables and cervix cancer

Variable1	Variable2	Coefficient correlation
Case–control	Menstrual disorder	0.882
	Conttaception_method	0.890
	Successful pregnancy	− 0.603
	Age first pregnancy	0.612
	Sexmate_patient_2group	0.51
	Age first sex	0.88
	Number of sex in month	0.823

**Table 6:** Correlation between demographic variables and cervix cancer

Variable1	Variable2	Coefficient correlation
Case–control	Residient_place	0.982
	Financial_status	0.935
	Marriage	0.964
	Education_status	0.934
	Alcohol	0.75

As shown in Table 5, Conttaception_method, Menstrul disorder, Number of sex in a month, the age of first sex has a positive correlation with cancer and successful pregnancies have negative correlation on cervix cancer.

As can be deduced from Table 6, Residient_place, Financial_status, Marriage_status, Education_status and Alcohol are associated with cancer.

#### Response to aim3: identifying important coefficients on cervical cancer in each category

According to the results obtained from Table 7, the most important coefficients in combination with other variables in each category from high to low can be seen in each category, for example, in the macronutrients category, phosphorus, selenium and zinc have the most effect on cancer, respectively.

**Table 7:** Important coefficients in each category with machine learning methods

Target category	Variable’s name	Coefficients of each variable	Performance of model
Important micronutrients identified by deep learning	Phosphorus (mg)	1	MSE: 0.036 R^2^: 0.855 AUC: 0.993 Accuracy:96.31
	Selenium	0.981	
	Iron (mg)	0.877	
	Zinc	0.860	
	Niacin (mg)	0.824	
	Thiamin (mg)	0.818	
	Folate	0.793	
	Vitamin B6 (mg)	0.789	
	Calcium (mg)	0.784	
	Potassium (mg	0.750	
	Cooper	0.721	
	Iodin	0.721	
	Chloride	0.712	
	VitaminD	0.688	
	Carotene	0.673	
Important macronutrients and other nutrient factors identified by deep learning	Polyunsaturated fatty acid	1.000	MSE: 0.016 R^2^: 0.935 AUC: 0.999 Accuracy:98.80
	Salt	0.963	
	Milk	0.868	
	Snacks	0.861	
	dietary fiber	0.860	
	WholeBread	0.824	
	Legumes	0.798	
	Yogurt	0.722489	
	Tot.N2g	0.712064	
	Tea	0.701140	
	Starch	0.671164	
	Sugar	0.663765	
	protein.g	0.586717	
Important sexual factor identified by decision tree	Age first sex Menstrual disorder Number of sex	Accuracy: 99.66	
Important demographic factors identified by decision tree	Marriage,education	Accuracy: 99.90	
Important medical examination factors identified by decision tree	Smear Exocervix Hpv-cat Wart Hpv-positive	Accuracy: 98.47	

## Discussion

Cervical Cancer has a high mortality rate in women and endangers their lives. Identifying the most important factors of cancer is a critical challenge in prevention strategy and can even be helpful in early diagnosis. Among the factors that not be overlooked is the influence of diet/nutrition. Therefore, in the present study, the factors affecting cervical cancer, especially dietary factors and vitamins, were studied. Also, non-nutritional factors affecting cervical cancer and phase were identified.

Our findings indicated that, Phosphorous, selenium, Iron and zinc have an impact in reducing the risk of cervical cancer and progression of phase, as well as salt, snacks and milk were determined as high risk food factors. dietary fiber, starch and Tot.N2.g also has a beneficial impact on cervical cancer and phase. Meat and vegetables as well have a reducing effect on phase progression. Seasonal and tree fruits also have a good effect on cancer and phase.

Similar and contradictory results have been reported in various studies, which are mentioned following. In Meta-analysis by Myung et al. reported that carotene was associated inversely with cervical cancer risk and vitamin A had no effect on cervical cancer risk [19]. In a meta-analysis by Cao et al. Vitamin C was significantly associated with cervical cancer reduction risk [20]. In study by Hosono et al. Vitamin D intake, in Guo et al. study α-carotene, β-carotene and vitamins E and C, in research of Manju et al. vitamin C, E had a preventive role in cancer [14, 21, 22]. Beneficial effects of fruits and vegetables on cancer prevention have been reported in some studies [23–25].

The effect of nutrients on HPV has been studied in several studies. A study in Sao Paulo reported that the consumption of papaya plays a preventive role against HPV infection [26]. In the Chih et al. study, the consumption of fruits, vegetables, yogurt, fish, tofu and meat was considered to be effective to decrease the risk of HPV [27]. The Result of Barchitta et al. study shows that a high intake of red and processed meats, dipping sauces, chips, and snacks with a low intake of olive oil in the Western diet, was related to a higher risk of HPV. In contrast, Mediterranean diet (MD), Consisting vegetables, legumes, fruits and nuts, cereals, fish, and a high ratio of unsaturated to saturated lipids had a lower risk of HPV [28].

Sedjo et al. reported high consumption of vegetables and carotenoid be beneficial in reducing HPV risk [29]. In the review of Koshiyama M publisher in 2019, multi-vitamin, vitamin A, vitamin C, vitamin D, vitamin E, papaya, Mediterranean diet, carotenoids, fruits, vegetables, legume, lycopene, green tea, folate, sulforaphane, polyphenol Flavonoids, polyunsaturated fatty acid, calcium (±) reported generally as main preventive and reductive factors against CC risk and introduced cigarette, Western diet and oleic acid as high-risk diets/nutrients. Also Mediterranean diet, papaya Vitamin-C, vegetables, carotenoids and fruits had a reductive effect on HPV infection [16].

Piyathilake et al. showed that folate has a significant inverse association with HPV infection [30]. A study by Giuliano et al. found a relationship between persistent HPV infection and low intake of vitamin C [26]. Sedjo et al. showed that vegetables and fruits and juices were associated with a reduction in the risk of HPV persistence [29]. In studies, the effect of a diet containing vegetables on cancer prevention and HPV was positive [24, 27, 31–33]. We did not find any correlation between nutrient intake and HPV in the present study.

Some research has studied the association of socioeconomic characteristics with cancer. In some studies, Age at first marriage, Number of deliveries and Contraceptive methods have been reported as important factors on cervical cancer [34]. In study of Nojomi et al. low marriage age, high prevalence of pregnancy, family history, contraceptive pills and Low age at first pregnancy associated with cervix cancer [35]. In the study by Vaisy et al. marriage at age below 16, marital status, married more than once, consumption of Protective factors were reported as influence factors and Contraceptive pill [36]. In contrast, in the study by Mohaghegh et al. multiple marriages and multiple sexual partners were significant, but smoking, diet, and being widowed or divorced, have no significant correlation with cancer [37]. Tadesse showed poverty, early marriage, and high parity influence factors were associated with cervical cancer [38]. In Ansari study, socio-economic factors were reviewed as key factors in cervical cancer, such as increasing age, education, knowledge, marital status, multiple sexual partners, financial status, using inappropriate clothes or having bad sanitary during menstruation, sexually activeness, HPV, post-menopausal bleeding, offensive vaginal discharge, having many pregnancies, pills and injections [39].

In our research, constipation method, menstrual disorder, number of sex in a month, the age of first sex and Sexmate_patient_2group has a positive correlation with cancer and successful pregnancies has a negative correlation with cervix cancer. Among demographic factors, Residient_place, Financial_status, Marriage_status, Education_status and Alcohol were correlated with cancer. Smear, hpv_sign_cat, wart and hpv positive also had important examination factors for cervix cancer diagnosis.

## Conclusions

With Developing a comprehensive strategy, cervical cancer can be effectively controlled. Discovery factor affecting cervical cancer facilitates prevention, diagnosis and treatment. As in the present study, all factors affecting the incidence of cervical cancer were investigated to develop an appropriate prevention protocol.

In research during recent years, antioxidant vitamins have attracted much attention in cancer prevention. Because they protect cells from oxidative DNA damage and enhance the immune system.

Based on the results of the present study, healthcare workers should educate women to consider vitamins and useful nutrition in their dietary regime to prevent the development of cervical cancer. Education about cervical cancer should offer to women, families and communities.

The influence of diet and nutrition mechanisms on cervical cancer is unknown. Further research is needed to clarify these mechanisms. Foods consumed by humans are widespread and the effects of all nutrition cannot be measured, it is recommended that other foods be considered in research. Most articles were epidemiological and clinical trials. There were few experimental studies in this regard. It is recommended more experimental studies will do. Otherwise, different nutrition/diets may have differing impacts on cancer in the different geographic places. Therefore, it is necessary that similar studies would be done in different countries in the future. In general, we must continuously avoid the consumption of large amounts of high-risk diets and nutrients, and at the same time continuously consume preventive and reductive diets and nutrients. Therefore, Lifestyle modifications, including attention to diet, social habits, sexual behavior and vaccination can greatly prevent cancer.

## Data Availability

The datasets generated and/or analysed during the current study are not publicly available but are available from the corresponding author on reasonable request.

## Declarations

### Permission and/or credit for reproduced images

All images in current manuscript were produced by authors of the manuscript.
